# Benefits of Surgical Management in Ileocecal Crohn’s Disease: A Review of the Past Decade (2020–2026)

**DOI:** 10.3390/medicina62050949

**Published:** 2026-05-13

**Authors:** Ion Balint, Roxana Zaharie, Vălean Dan, Emil Moiș, Călin Popa, Andra Ciocan, Nadim Al-Hajjar, Florin Zaharie

**Affiliations:** 1 Department of Surgery, University of Medicine and Pharmacy “Iuliu Hațieganu”, 400012 Cluj-Napoca, Romania; dr.ion.balint@gmail.com (I.B.); valean.d92@gmail.com (V.D.); drmoisemil@gmail.com (E.M.); calinp2003@yahoo.com (C.P.); andra.ciocan10@gmail.com (A.C.); na_hajjar@yahoo.com (N.A.-H.); florinzaharie@yahoo.com (F.Z.); 2 Regional Institute of Gastroenterology and Hepatology “Octavian Fodor”, 400162 Cluj-Napoca, Romania

**Keywords:** ileo-cecal Crohn, early surgery, Kono-S anastomosis, pediatric surgery, quality of life, morbidity, step-up approach

## Abstract

*Background and Objectives*: The management of localized ileocecal Crohn’s disease (CD) is undergoing a significant paradigm shift from traditional “step-up” medical escalation toward proactive early surgical intervention. With the evolution of surgical therapies as well as various minimally invasive procedures, as well as a better understanding of inflammatory bowel diseases, surgery is playing a more important role in the treatment of inflammatory bowel disease. One of the most common occurrences in Crohn’s disease, the ileocecal localization can present with a lot of dilemmas regarding the optimal treatment in both adult patients and pediatric patients alike. One of the biggest challenges remains the decision between early surgery and continuous biological treatment, which can prove a challenge from multiple standpoints ranging from cost-efficiency to recurrence rate. This review highlights the latest changes in surgical management in ileocecal Crohn’s disease, focusing primarily on the anastomotic type, comparison with biological therapy, early aggressive surgery and pediatric surgery. *Materials and* *Methods*: After respecting the review criteria, 16 articles were included in our study, which emphasize the importance and the recent trends in the surgical management of the ileocecal disease. *Results*: All 16 articles met criteria for good quality, suggesting a low risk of bias, focusing primarily on early surgery, the role of Kono-S anastomosis as well as pediatric considerations. *Conclusions*: While the choice of the Kono-S anastomosis remains debatable, significant progress has been made in terms of early surgery which improves the long-term outcomes in patients while minimizing the risk of morbidity and mortality.

## 1. Introduction

Crohn’s disease (CD) represents one of the primary entities within the range of the inflammatory bowel diseases (IBDs), being characterized by transmural inflammation that can affect any segment of the digestive tract, most commonly involving the terminal ileum and the proximal colon, which involves up to 30% of cases. It presents with a high degree of morbidity and due to its interplay between genetic, environmental and microbiotic factors, its incidence is increasing around the world, affecting not only developed countries but also developing countries, thus posing challenges to healthcare systems around the world [1].

Presenting with a high variability of complications as well as a complex treatment, it is mandatory to ensure an adequate diagnosis as well as pinpointing the exact complications, while preserving a satisfactory quality of life for every patient with CD [2]. This can be a challenge especially in cases which involve the ileocecal region since there is a thin line between requirement of surgery versus medical therapy, and more so in uncomplicated cases. Even though most complicated cases will require surgery, the challenge arises in uncomplicated cases, its management remaining open for discussion [3,4]. The molecular-immunological mechanism of CD involves a dysfunctional immune response triggered by an interplay between genetic susceptibility, environmental factors, and altered gut microbiota. This results in the breakdown of the intestinal epithelial barrier and the recruitment of inflammatory cells, such as macrophages and T-cells, which release high concentrations of pro-inflammatory cytokines like tumor necrosis factor-alpha (TNF-alpha). This sustained immune activation leads to characteristic tissue damage, including deep ulcerations and the “creeping fat” phenomenon where mesenteric adipose tissue migrates toward inflamed bowel segments [1,2,3].

The landscape of management for CD localized to the ileocecal region has undergone a transformative evolution over the last decade, reflecting broader advances in both medical therapy and surgical strategy. Traditionally, the therapeutic algorithm followed a rigid “step-up” approach in which patients were initially treated with conventional medical therapies such as corticosteroids, immunomodulators, and later biologic agents, with escalation occurring only after failure of preceding treatments [5]. Within this framework, surgery was viewed mostly as a salvaging procedure, or as means to treat potential life-threatening complications [5,6]. It was typically reserved for patients who experienced refractory disease despite maximal medical therapy or who developed irreversible structural complications of chronic inflammation, including fibro-stenotic strictures causing obstructive symptoms, penetrating disease with internal fistulae, or intra-abdominal abscess formation [5,6,7]. Consequently, surgical intervention was often performed late in the disease course, when cumulative bowel damage and systemic disease burden had already significantly progressed to the point in which conventional therapy seemed redundant [8].

Recent studies, however, have shown a substantial shift towards “early surgical intervention”, especially in patients with localized disease. In addition, in carefully selected patients with limited disease distribution, early resections can offer outcomes superior to long-term biological treatment, maintaining adequate disease control, with adequate levels of quality of life as well as minimizing costs [9,10,11]. This shift reflects the fact that surgery could be considered the mainstay of treatment in inflammatory bowel diseases, most notably Crohn’s disease, as it can be considered a proactive method of treatment which may influence the trajectory of the disease [9].

This evolving treatment paradigm also aligns with the concept of preventing cumulative bowel damage, which is increasingly recognized as a key determinant of long-term disability in Crohn’s disease. By intervening earlier in the disease course—before chronic inflammation leads to irreversible fibrotic remodeling and complex penetrating complications—surgical resection may interrupt the cycle of inflammation and tissue damage, potentially modifying the natural history of ileocecal CD. As a result, early surgical intervention is now being increasingly discussed within multidisciplinary care frameworks involving gastroenterologists, colorectal surgeons, and radiologists, with treatment decisions tailored to individual patient characteristics, disease phenotype, and personal preferences [12].

The primary aim of this narrative review with a structured literature search is to evaluate the evolving role and clinical benefits of proactive surgical management in ileocecal Crohn’s disease, specifically comparing early intervention strategies with traditional medical escalation. To provide a comprehensive assessment of this paradigm shift, this review synthesizes evidence across several critical therapeutic endpoints, including the rates of endoscopic and clinical recurrence and the long-term risk of reoperation. Furthermore, we examine the impact of surgical timing on health-related quality of life and the cost-effectiveness of early resection compared to chronic biological therapy. Special emphasis is placed on the achievement of therapy-free remission and pediatric-specific outcomes, such as the restoration of growth velocity and clinical remission in young patients. By structuring the analysis around these defined metrics, this review seeks to provide a data-driven framework for optimizing the timing of surgical intervention within a multidisciplinary care setting.

## 2. Materials and Methods

Systematic research of the primary databases (Embase, PubMed, Scopus) was performed, without language restrictions, with the search strategy focusing primarily on identifying studies evaluating ileocecal resection outcomes in inflammatory bowel diseases. The research was performed according to the Preferred Reporting Items for a Systematic Review and Meta-analysis of Individual Participant Data Criteria. The keywords used were as follows: “ileo-cecal resection, Crohn’s disease, prognostic and outcome, follow-up, early surgery”.

The systematic search focused on identifying clinical trials, meta-analyses, systematic reviews, and retrospective cohort studies evaluating ileocecal resection outcomes. The selection process was focused on the last decade (January 2020–January 2026) and was divided into two stages: the primary stage focused on reading the titles and abstracts, and the secondary stage focused on analyzing the contents of the articles for further reviewing. Articles that were selected after this two-stage process were included in this review. The selected articles are summarized into a table, highlighting the year of the article, primary authors, type of study, design of the study, primary outcomes and highlights of the study, as well as limitations of the study. No automation tools were utilized; all authors were involved in the selection process. Due to the clinical and methodological heterogeneity of the included studies, a structured systematic narrative review was conducted to synthesize the findings.

To address the requirement for methodological transparency, the research objective was formalized using the PICO (Population, Intervention, Comparator, and Outcome) framework:

• Population (P): The study population consists of both adult and pediatric patients diagnosed with localized ileocecal Crohn’s disease (CD).
• Intervention (I): The primary intervention is “early surgery,” which for the purpose of this review is operationally defined as primary ileocecal resection (ICR) performed within 30 days of diagnosis or as a proactive intervention before the development of irreversible structural complications.
• Comparator (C): The intervention is compared against the traditional “step-up” medical approach—including the use of corticosteroids, immunomodulators, and biologic agents—or delayed/salvage surgery performed after medical therapy failure.
• Outcome (O): The primary outcomes measured are clinical and endoscopic recurrence rates and postoperative complication rates. Secondary outcomes include health-related quality of life (HRQoL), long-term healthcare costs, and metabolic/growth improvements in pediatric patients.

To ensure consistency across the heterogenous studies, the following definitions were applied.

• Early surgery: Primary surgical resection performed as a first line or early line of strategy, specifically defined as intervention within 30 days of diagnosis.
• Localized disease: Crohn’s disease limited to the ileocecal area.
• Uncomplicated disease: Absence of fibrostenotic strictures or penetrating complications such as fistulae or intra-abdominal abscesses

### 2.1. Search Strategy

**Figure 1:**
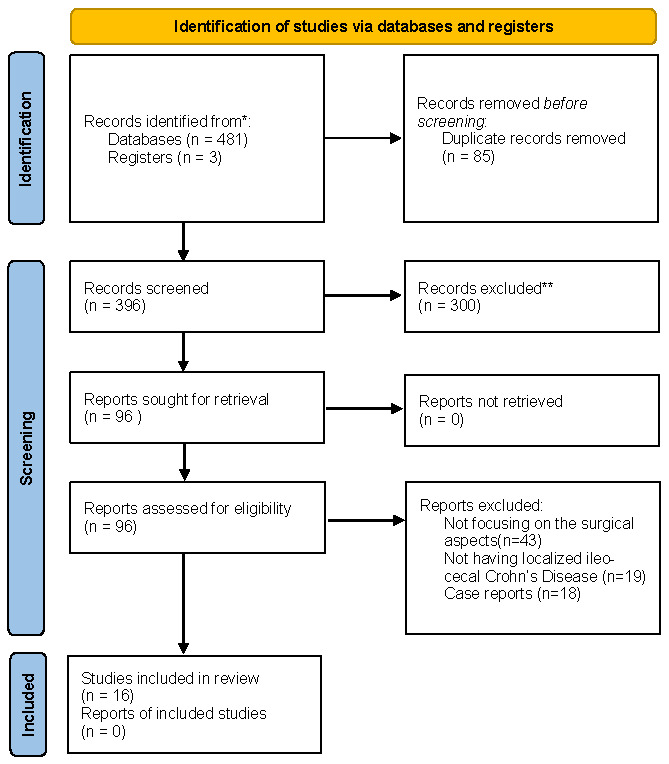
PRISMA diagram. * Databases used were mentioned in the Section 2, ** Removed based on exclusion criteria.

A total of 481 records were identified across the three databases. Following the removal of 85 duplicates, 396 unique articles were screened (Figure 1). During the primary screening phase (title and abstract), 300 articles were excluded due to a lack of focus on ileocecal disease or for being case reports. The remaining 96 articles were assessed for full-text eligibility. Following a comprehensive review, 80 articles were excluded for not meeting the selected PICO criteria or lacking primary outcome data, resulting in 16 articles that met all inclusion criteria for the final review (Table S1).

### 2.2. Included Studies

The studies included are listed in Table 1. All studies were published between 2020 and 2026, the majority being published after 2023 (12/16 studies—75%). Primarily, studies focused on improvement of surgical techniques, postoperative recurrence rate, and timing of surgical intervention, as well as benefits of early surgery, considering various approaches, in both adult and pediatric patients. In addition, the benefits of early surgery in comparison with early immunomodulation therapy were highlighted.

**Table 1:** Articles included in the study.

Author et al.	Year	Article Title	Cases	Study Type and Design	Key Results (Outcomes/Safety)
Oldenburg et al. [13]	2026	Ileocaecal resection versus infliximab for ileal Crohn’s disease: 10-year follow-up of the LIR!C trial	134	Cohort study; retrospective 10-year follow-up of the LIR!C RCT.	10-year therapy-free remission: 35.8% vs. 13.2% (*p* = 0.004). Strong age-dependent benefit.
Flaifel et al. [14]	2025	The outcomes of robotic ileo-colic resection in Crohn’s disease compared with laparoscopic and open surgery: a meta-analysis and systematic review	5760	Meta-analysis; systematic review of RIR vs. LIR/OR.	Robotic approach had longer operative times (226 min) but shorter hospital stays and fewer complications than open surgery.
Avellaneda et al. [15]	2025	Long-term comparative outcomes after ileocecal resection for inflammatory versus complicated Crohn’s disease. A multicenter, retrospective study (Crohn’s-Urg)	2013	Cohort study; international multicenter retrospective (Crohn’s-Urg).	Comparable long-term recurrence between inflammatory and complicated phenotypes.
Baloyiannis et al. [16]	2024	The Reduction of Anastomosis-Related Morbidity Using the Kono-S Anastomosis in Patients with Crohn’s Disease: A Meta-Analysis	913	Meta-analysis; 8 studies evaluating the Kono-S hand-sewn anastomosis.	Reduced leak risk (OR = 0.34) and reoperation (OR = 0.12). No change in overall morbidity.
Madaffari et al. [17]	2024	Early Surgical Resection in Pediatric Patients with Localized Ileo-Cecal Crohn’s Disease: Results of a Retrospective Multicenter Study	29	Pediatric cohort study; evaluating early surgical intervention.	83.7% clinical remission at 1 yr. Median SES-CD decreased from 12 to 0 (*p* < 0.0001).
Tyrode et al. [18]	2024	KONO-S Anastomosis Is Not Superior to Conventional Anastomosis for the Reduction of Postoperative Endoscopic Recurrence in Crohn’s Disease	366	Randomized controlled trial; multicenter prospective randomized study.	No significant difference in 12–18 m endoscopic recurrence (31.6% Kono-S vs. 33.3% SSA).
Alibert et al. [19]	2024	Does Kono-S Anastomosis Reduce Recurrence in Crohn’s Disease Compared with Conventional Ileocolonic Anastomosis? A Nationwide Propensity Score-matched Study from GETAID Chirurgie Group [KoCoRICCO Study]	433	Propensity score analysis.	Kono-S did not reduce risk of endoscopic recurrence ≥ i2 compared to SSA (47.5% vs. 44.3%).
Agrawal et al. [20]	2023	Early ileocecal resection for Crohn’s disease is associated with improved long-term outcomes compared with anti-tumor necrosis factor therapy: A population-based cohort study	1279	Retrospective cohort; propensity-weighted Danish register analysis (2003–2018).	ICR associated with 33\% lower risk of composite adverse outcomes vs. anti-TNF (aHR = 0.67).
Beelen et al. [21]	2023	Impact of timing of primary ileocecal resection on prognosis in patients with Crohn’s disease	822	Cohort study; retrospective multicenter assessment.	Postoperative recurrence was not affected by timing of the ileocecal resection.
Sarikaya et al. [22]	2023	Disease course and treatment outcomes of Crohn’s disease patients with early or late surgery—A Danish nationwide cohort study from 1997 to 2015	2483	Nationwide cohort study.	Early resection had a lower cumulative risk of reoperation and a lower cumulative exposure to immunomodulators.
Mineccia et al. [23]	2022	Has the Removing of the Mesentery during Ileo-Colic Resection an Impact on Post-Operative Complications and Recurrence in Crohn’s Disease? Results from the Resection of the Mesentery Study (Remedy)	326	Retrospective cohort.	Resection of the mesentery does not reduce endoscopic and recurrence rate.
Dreznik et al. [24]	2023	Recurrence rates following ileo-colic resection in pediatric patients with Crohn’s disease	35	Single-center retrospective study.	Lower postoperative recurrence following ileo-colic resection especially in the first years of surgery.
Pak et al. [25]	2021	Short- and long-term outcomes of laparoscopic versus open ileocecal resection for Crohn’s disease: a propensity score-matched study	348	Cohort study; propensity -score-matched comparison of LICR vs. OICR.	LICR reduced complications (14\% vs. 32\%) and hospital stay (8 days vs. 13 days).
Kelm et al. [26]	2021	Early Ileocecal Resection Is an Effective Therapy in Isolated Crohn’s Disease	103	Cohort study; retrospective single-center assessment.	Early ICR reduced the need for postoperative immunosuppressive medication.
Luglio et al. [27]	2020	Surgical Prevention of Anastomotic Recurrence by Excluding Mesentery in Crohn’s Disease: The SuPREMe-CD Study—A Randomized Clinical Trial	113	Randomized controlled trial; Kono-S vs. conventional stapled side-to-side.	Kono-S group showed significantly lower endoscopic recurrence (*p* < 0.05) and clinical recurrence at 12–18 months.
Hirten et al. [28]	2020	Anastomotic Ulcers After Ileocolic Resection for Crohn’s Disease Are Common and Predict Recurrence	182	Retrospective cohort.	In multivariable analysis, anastomotic ulcers were associated with disease recurrence (adjusted hazard ratio [aHR] 3.64; 95% CI, 1.21–10.95; *p* = 0.02). Sixty-six subjects with anastomotic ulcers underwent a second colonoscopy, with 31 patients (79.5%) having persistent ulcers independent of medication escalation.

### 2.3. Quality Assessment and Certainty of Evidence

To evaluate the methodological quality of the included studies, two reviewers independently utilized the Newcastle–Ottawa Scale (NOS) for observational cohort studies and the Cochrane Risk of Bias tool (RoB 2) for randomized controlled trials (RCTs). Discrepancies were resolved through consensus. For observational studies, quality was categorized as high (7–9 stars), fair (5–6 stars), or poor (<5 stars). Furthermore, the certainty of the body of evidence was evaluated using the Grading of Recommendations Assessment, Development and Evaluation (GRADE) framework, which classifies evidence as high, moderate, low, or very low based on risk of bias, inconsistency, indirectness, and imprecision.

## 3. Results and Discussion

### 3.1. General Considerations

The quality assessment of the included studies is highlighted in Table 2.

It has been widely debated that the step-up approach in inflammatory bowel diseases is the most suitable option, with surgery playing more of a salvaging role, managing end-stage complications, such as symptomatic stenoses, fistulae, and abscesses, thus presenting with a high postoperative morbidity and complication rate. Historical data consistently indicated that despite the advent of biologic therapies in the late 1990s, the lifetime risk of surgery remained substantial, with approximately 70% to 80% of patients requiring at least one resection during their disease course. Pre-2020 clinical guidelines, such as the 3rd European Evidence-based Consensus on Crohn’s Disease (2016/2017), typically advocated for a ‘step-up’ medical approach. In this model, surgery was primarily reserved for patients refractory to corticosteroids and immunomodulators or those presenting with end-stage mechanical complications like fibrostenotic obstructions and penetrating disease. Furthermore, the ECCO-ESCP Consensus (2018) emphasized that while surgery is often required for medically refractory disease, the focus of ileocecal CD was traditionally on salvaging rather than primary intervention [29]. In this model, surgery was reserved for patients who were refractory to corticosteroids and immunomodulators or those presenting with end-stage mechanical complications like fibrostenotic obstructions and penetrating disease. However, with the increasing trend of minimally invasive surgery in the last two decades, as well as a tendency of early diagnosis and prognosis of the disease, there has been a shift towards using surgery as a mainstay treatment especially in early lesions. This shift has been highlighted by the LIR!C Study Group published by Ponsioen et al. [30], who in a multicentric trial developed in 2017, highlighted that laparoscopic resection in patients with limited ileocecal disease can be considered as a reasonable treatment to infliximab, without reducing quality of life. This has paved the way for highlighting the importance of surgery as a main-stay treatment in limited, non-stricturing Crohn’s disease. Further trials have managed to reproduce the results, although a certain number of studies have contested the statement.

Another significant turning point in the preceding decade was the POCER trial (2015), which introduced a proactive “treat-to-target” paradigm for postoperative management. This landmark study demonstrated that personalized care based on early endoscopic monitoring (at 6 months) and risk-stratified medical prophylaxis could significantly reduce the rate of clinical and endoscopic recurrence compared to standard care. Together, these foundational research efforts established the feasibility of early surgical intervention, transitioning the role of the surgeon from an emergency provider to a key member of the multidisciplinary team involved in primary disease management [31].

The findings of this review were evaluated using the GRADE framework to stratify the strength of current clinical evidence. Evidence of high certainty supports the primary conclusion that early surgical timing—specifically intervention within 30 days of diagnosis—significantly reduces the long-term risk of reoperation and cumulative medication exposure. This conclusion is robustly corroborated by high-quality meta-analytical data and nationwide cohorts involving over 8000 cases, which consistently demonstrate that minimally invasive robotic and laparoscopic approaches further minimize postoperative morbidity compared to traditional open surgery. Evidence of moderate certainty identifies a transformative benefit in 10-year therapy-free remission for early surgery-first strategies compared to conventional biologic escalation, as demonstrated in the longitudinal follow-up of the LIR!C cohort. Similarly, moderate-certainty evidence supports the use of early resection in pediatric populations to facilitate significant “catch-up” growth and metabolic restoration. Conversely, evidence regarding specialized technical interventions, such as the Kono-S anastomosis and radical mesenteric resection, remains of low certainty. This downgrade is primarily due to serious inconsistencies between early single-center randomized trials and subsequent large-scale, multicenter propensity score-matched studies, which failed to replicate initial reductions in endoscopic recurrence. Consequently, while the timing of surgery is supported by high-quality data, the selection of specific anastomotic techniques remains a secondary consideration that requires further standardized validation. The current data is synthesized in Table 3.

### 3.2. Early Surgery Versus Medical Therapy in Ileocecal Crohn’s Disease

The clinical management of Crohn’s disease (CD), particularly when localized to the ileocecal region, has been defined by a multi-decade tension between surgical and pharmacological paradigms. Traditionally, the terminal ileum and cecum have been the most frequent sites of involvement, accounting for approximately 25 to 30 percent of all diagnosed cases [32]. In the pre-biologic era, treatment was largely reactive, utilizing a “step-up” approach that began with 5-aminosalicylates or sulfasalazine, progressed to corticosteroids for remission, and eventually employed immunomodulators such as azathioprine or methotrexate for maintenance [33]. Within this traditional framework, surgery was viewed as a treatment failure “last resort” reserved for patients who developed life-threatening complications like complete mechanical bowel obstruction, free perforation, or refractory intra-abdominal abscesses [34].

Contemporary clinical discourse now centers on the “window of opportunity”, a period early in the disease course when the bowel wall remains predominantly characterized by inflammatory activity rather than irreversible structural damage like fibrosis or complex fistulization. Systematic reviews and meta-analyses published between 2023 and 2026 have increasingly supported the role of early ileocecal resection (ICR) as a proactive, rather than reactive, therapeutic option for patients with uncomplicated, localized disease [35,36,37,38].

The studies that focused on the differences between early surgery and medical therapy are highlighted in Table 4. All studies were observational and retrospective, primarily focusing on the impact of early ICR on a population ranging from 103 cases to 2483 cases.

The continuation of the Lir!c trial, highlighted by Oldenburg et al., is a 10-year follow-up of the original RCT published in 2017 [13]. The primary endpoint of the LIR!C trial was health-related quality of life (HRQoL), measured using the Inflammatory Bowel Disease Questionnaire (IBDQ) at 12 months. The results indicated that quality of life was comparable between the two groups, demonstrating that surgery is not inherently “worse” for a patient’s daily functioning than chronic medical therapy. While the surgical group experienced an initial period of recovery, the long-term stabilization of symptoms was equivalent to that achieved by Infliximab (IFX). However, long-term follow-up of the trial participants yielded further results. By five years, half of the initially randomized patients in the Infliximab group eventually required a surgical resection. In contrast, 42% of the patients in the resection group required no medical treatment during the follow-up, although half of them received prophylactic immunomodulators. The most recent data provides a 10-year outlook on the current cohort, with the emphasis on “therapy-free remission”. When comparing the ileocecal resection group with the infliximab group, the therapy-free remission rate was 35.8% vs. 13.2%. Although no statistically significant differences were highlighted between the overall clinical remission rates, there were significant differences between the remission rates in 20-year-old patients (54% ICR vs. 24% IFX). In addition, the data demonstrated that early surgery results in lower hospitalization costs over the long term, compared to biological therapy, because the cost of one-time laparoscopic procedure with a short hospital stay is offset by the usage of long-term biological therapy.

This statement is also supported by Kelm et al., who in 2021 highlighted in a retrospective analysis of 103 patients that early ICR reduces the need for postoperative medication during a two-year follow-up [26]. In addition, patients with early ICR required a decreased amount of medical therapy postoperatively. Furthermore, within 2 years of surgery, only one patient required therapy escalation from the early ICR group compared to twenty patients (27%) from the group with patients who underwent surgery at a later stage. This is also complemented by the work of Agrawal et al., who in 2023 analyzed a population-based cohort of 1279 patients. Their propensity-weighted Danish register analysis demonstrated that ileocecal resection was associated with a 33% lower risk of composite adverse outcomes compared with anti-TNF therapy (HR = 0.67). Such data indicates that the benefits of surgery extend beyond mere symptom control, offering a safer long-term profile regarding disease-related complications [20].

One of the more pivotal studies was the Danish nationwide cohort study published by Sarikaya et al., who in 2023 gathered 2483 patients over the span of 18 years [22]. It highlighted the benefits of early surgery (within 29 days of diagnosis) compared to intermediate (30–180 days) or late surgery (over 180 days). The patients who underwent resection within 29 days had a 5-year reoperation risk of 16.5% compared to 18.2% and 21.2% respectively in the other two groups. In addition, patients with later surgery had fewer initial hospitalizations, and those in the delayed surgery group had a higher cumulative use of immunomodulators following their initial operation. Thus, early surgical intervention (within 29 days of diagnosis) can offer better long-term prognosis regarding reoperation, and also reduces reliance on immunomodulators.

However, the debate regarding “early” vs. “late” surgery continues. Beelen et al. (2023), in a retrospective multicenter assessment of 822 cases, found that while postoperative recurrence was not significantly affected by the timing of the primary ileocecal resection, patients operated on within 30 days of diagnosis had a 14% lower reoperation rate compared to those who underwent surgery after 12 months. This implies that while the biological recurrence of the disease might be inherent, surgical complexity and subsequent surgical failures are mitigated by early intervention [21].

### 3.3. Evaluating the Kono-S Anastomosis in Prevention of Recurrence

Recent insights into the pathophysiology of CD have highlighted the mesentery as a potential driver of the inflammatory pathway. Strategies aimed at resecting the associated mesentery (the “Kono-S” or “radical mesenteric resection” approach) are being explored as avenues to further reduce the risk of postoperative recurrence, as the mesentery contributes to a pro-inflammatory environment through complex molecular interactions with the intestinal wall. The Kono-S anastomosis, an antimesenteric hand-sewn functional end-to-end anastomosis for Crohn’s disease, was first performed by Dr. Toru Kono in Japan in 2003 to reduce postoperative recurrence [39]. It gained international attention for its superior long-term results, showing low surgical recurrence rates (approximately 0–4%) by utilizing a supporting column to maintain luminal width. The technique has been adopted worldwide, with many studies praising its efficiency in lowering the recurrence rate as well as maintaining low postoperative comorbidities. Table 5 shows a selection of reviewed studies which focus on the effectiveness of Kono-S anastomosis.

One of the primary randomized controlled trials focusing on the recurrence rate post Kono-S anastomosis was the SuPREMe-CD trial, published by Luglio et al. in 2020 [27]. Over a course of 113 cases, it demonstrated a dramatic reduction in endoscopic recurrence at one year (22.2% vs. 62.8% in stapled side-to-side, *p* = 0.001). In addition, the trial found that Kono-S anastomosis has a lower clinical recurrence rate (8–18% vs. 18–30.2% in the control group) as well as associating the technique with a wider lumen, offering a significantly better option compared with the traditional techniques. These findings are supported by the meta-analysis published by Baloyiannis et al., who in 2024 evaluated 913 patients over eight studies, showcasing a substantially reduced risk of anastomotic leak (OR = 0.34) and reintervention (OR = 0.12) in patients with CD using the Kono-S technique, despite not being superior in terms of overall morbidity (OR = 0.69), showcasing an increased risk of postoperative SSI (OR = 1.69) [16].

However, more recent and larger-scale studies have offered a more tempered view. The KoCoRICCO study (Alibert et al., 2024), a nationwide propensity score-matched study from the GETAID Chirurgie group, failed to replicate the previous findings, and found that Kono-S did not significantly reduce the risk of endoscopic recurrence (larger than i2—Rutgeerts score) compared to conventional anastomosis (47.5% vs. 44.3%, OR = 1.41) [19]. Similarly, Tyrode et al. reported in a multicenter prospective randomized control trial of 366 patients that there was no significant difference in endoscopic recurrence at 12–18 months (31.6% Kono-S vs. 33.3% side-to-side anastomosis). In addition, at 6–12 months, endoscopic postoperative recurrence rate did not differ significantly between the groups (56.7% Kono-S vs. 49.1% in control group) [18].

Despite showcasing conflicting findings regarding postoperative and endoscopic recurrence, the safety profile of Kono-S remains uncontested, thus remaining a valid technique associated with reduced risk of surgical complications. While all aforementioned studies show low rates of anastomotic leak, more recent studies focus on developing mechanical versions of the Kono-S anastomosis to further decrease the operative time.

### 3.4. Surgical Modalities in Ileo-Colic Resections in Crohn’s Disease

In the contemporary setting of Crohn’s disease, surgical modalities for ileo-colic resection have evolved from traditional open laparotomy toward minimally invasive surgery (MIS), with laparoscopic-assisted resection now established as the gold standard due to its association with faster recovery, reduced postoperative ileus, and lower incisional hernia rates. More recently, robotic-assisted ileo-colic resection has emerged as a viable alternative, offering superior visualization and dexterity in complex cases involving dense adhesions or fistulae, though it is often characterized by longer operative times compared to conventional laparoscopy [40]. Beyond the surgical access route, the technical configuration of the anastomosis remains a critical area of investigation. Furthermore, bowel-sparing procedures, including stricturoplasty, may be considered in selected patients to avoid short bowel syndrome, particularly in those with recurrent disease [41]. The choice of surgical modality is therefore multifactorial, influenced by disease phenotype, prior surgical history, patient comorbidities, and surgeon expertise, underscoring the need for individualized, multidisciplinary decision-making in the management of ileo-colic Crohn’s disease [40,41].

The success of early surgical intervention is linked to advancements in surgical technique and perioperative care. The transition from large-incision laparotomy to minimally invasive laparoscopic resection has been a primary factor for improved patient acceptance and reduced morbidity, as well as obtaining better results with a lower rate of recurrence. Although it has been widely accepted that laparoscopic resections are the gold standard for localized CD, in the context of early surgery, where the disease is purely inflammatory, the procedure is less technically demanding, due to the fact that inflammation without the presence of complications such as fistulae, adhesions, or abscesses will often result in a shorter operative time and lower rates of conversion. There are, however, some critical factors that need to be addressed regarding the safety and the outcome of surgery. Anastomotic configuration plays an important role that can provide a short-term impact by minimizing the risk of anastomotic leaks, as well as long-term in the prevention of recurrence, as mentioned above. Recent surgical research has also focused on the role of the mesentery in postoperative recurrence. The “creeping fat” phenomenon, where mesenteric adipose tissue wraps around the inflamed bowel, is a hallmark of Crohn’s disease [42]. There is an ongoing debate regarding whether “extended” mesenteric excision (removing more of the regional lymph nodes and mesentery) is superior to “limited” excision [43]. Table 6 showcases the current selection of articles regarding the surgical modalities and technical advancements in ileo-colic resections for Crohn’s disease.

Minimally invasive approaches have demonstrated clear superiority in the past decade in terms of recovery, complications rates and postoperative morbidity rate. In a cohort study highlighted by Pak et al., in 2021, using a propensity score-matched study of 348 cases, it was shown that laparoscopic ileocecal resection (LICR) presented with a significantly lower postoperative complication rate compared to open surgery (14% vs. 32%) as well as shorter hospital stay (8 days vs. 13 days), while providing comparable long-term recurrence-free survival, exceeding 90% in both cohorts [25]. Furthermore, there were no differences regarding postoperative comorbidities such as obstruction, although some certain risk factors such as malnutrition and penetrating disease need to be taken into consideration. However, one of the primary conclusions was that despite the improvement in short-term recovery sustained by the laparoscopic approach, the long-term management remains proportional to the disease’s natural history rather than the surgical approach.

The highlight of the past decade, however, remains robotic surgery, as highlighted by Flaifel et al., in 2025, through a meta-analysis consisting of 5760 cases, out of which 369 patients underwent robotic surgery [14]. It concluded that despite the longer mean operative time (226 min), it is associated with fewer postoperative complications as well as a shorter hospital stay compared to open surgery. Furthermore, robotic resections have shown a comparable outcome to the laparoscopic approach, apart from mean operative time. Another advantage is that it facilitates intracorporeal anastomosis, which may limit postoperative complications.

Another important factor in the postoperative outcome of surgery in ileocecal resections for Crohn’s disease remains the management of mesentery. While the Kono-S anastomosis primarily focuses on mesenteric exclusion, some studies have emphasized the role of mesenteric resection. The REMEDY study, published by Mineccia et al. in 2022, on a cohort of 236 patients, however, demonstrates that the resection of the mesentery does not significantly reduce endoscopic or clinical recurrence rates [23]. This may suggest that while mesenteric inflammation is a marker of disease activity, its removal may not be the optimal approach in terms of lowering recurrence rates. Endoscopic and ultrasonographic recurrences were similar in both groups (44.6% and 40.4% in groups with mesenteric resections vs. 46.7% and 41.2% in groups without mesenteric resections), without any statistically significant differences. Therefore, there is still an ongoing debate whether diseases with a mesenteric-oriented flare might benefit from aggressive resection.

Another important surgical outcome that has been debated remains the presence of anastomotic ulcers post ileocecal resections in CD. In a study published by Hirten et al. in 2020, which consisted of 182 patients who underwent ileo-colic resection, anastomotic ulcers were present in over 50% of cases (95 subjects), without associating any risk factor with ulcer development [28]. However, the presence of ulcers was associated with a significantly higher risk of clinical recurrence (OR = 3.64, CI: 1.21–10.95, *p* = 0.02), which highlights the importance of early postoperative endoscopic monitoring of the patients. It is also worth mentioning that 31 patients have shown persistent ulcers independent of medication escalation at second colonoscopy. This outcome can influence surgical decision and timing in the management of patients.

It is also worth noting the importance of safety in surgery, which is a priority for patients and clinicians alike; thus, maintaining a low rate of complications is paramount. Flaifel et al. also reported low rates regarding complications (9% in postoperative ileus, 7% in wound complications with a readmission rate of 13%), while other cohort studies reported similar results in previous years [14].

### 3.5. Role of Ileocecal Surgery in Pediatric Crohn’s Disease

Surgical management in pediatric patients requires a tailored approach, focusing on maintaining adequate nutrition for a proper developmental plan in children, as well as balancing the long-term impact of medication. Historically, the indications for surgery in children mirrored those in adults, focusing primarily on complications. However, with the recent developments in minimally invasive surgery, and all the methods of preserving an adequate bowel-length, surgery is playing a more important role.

Although there is a scarcity of data regarding the role of surgical resections in pediatric cases, the past decade highlighted valuable data providing its effectiveness. Madaffari et al. (2024), in a retrospective multicenter study of 29 pediatric cases, reported an 83.7% clinical remission rate at one year following early surgical resection for localized ileocecal CD [17]. A notable outcome in this study was the drastic decrease in the Simple Endoscopic Score for Crohn’s Disease (SES-CD), which dropped from a median of 12 preoperatively to 0 postoperatively (*p* < 0.001) This immediate reduction in inflammatory burden is critical for pediatric patients, as it often leads to a “catch-up” growth phase and improved nutritional status. However, while surgery is effective, early-age diagnosis remains a risk factor for early recurrence, as seen in over 16% of cases. Surgery also maintains a safety profile in pediatrics, with reasonable complication rates. Thus, in pediatric cases with limited, localized disease, early resection should be considered one of the front-row strategies that can improve quality of life. Moreover, these findings are supported by Glenisson et al. (2024). In a retrospective study of 43 pediatric patients, researchers found high efficacy for surgical management, with clinical remission rates of 84% at one year and 65% at 2.3 years. The postoperative recurrence rate was 16.3% at one year, rising to 35% by the end of the follow-up period [44].

These findings are also supported by Dreznik et al. (2023), who highlighted that postoperative recurrence rates in pediatric patients (35 cases) were significantly lower in the first years of surgery compared to historical cohorts [24]. This improvement is attributed to closer postoperative surveillance and the early initiation of medical prophylaxis where indicated. In pediatric cases, surgery is often used as a tool to avoid the long-term metabolic and developmental side effects of chronic steroid use and high-dose biologics. In contrast, Spencer et al. showcased an endoscopic recurrence rate of 46% at 2 years, and a histological recurrence of 44% in patients with endoscopic remission over a cohort of 78 patients. However, the results suggest that children would benefit from postoperative monitoring and early intervention and prophylaxis.

One particularly important aspect was highlighted by Weigl et al. in 2024, focusing on the metabolic benefits of surgery. In a cohort of 29 patients, the study demonstrated that early ICR leads to significant short-term improvements in growth. Postoperative z-scores for weight (improved from 1.78 to 0.77), BMI (1.08 to 0.22), and height (0.88 to 0.66) all showed significant gains (*p* < 0.001) [45].

Some authors focused on the efficiency of various anastomotic types in pediatric surgery. Dotlacil et al. (2024) investigated the usage of Kono-S anastomosis specifically in children. In a cohort of 25 participants, results showed good short-term safety with no surgical recurrence observed at a median follow-up of 29 months and a low endoscopic rate at 6 months (26%) [46]. Dipasquale et al. (2025) focused on the effect of different anastomotic configurations (end-to-end, side-to-side, and Kono-S). It found that no specific type of anastomosis was associated with an increased risk of endoscopic recurrence, suggesting that Kono-S and side-to-side techniques are safe alternatives to traditional end-to-end methods in the biologic era [47]. Similarly, Avellenda et al. highlighted that while complicated Crohn’s disease is more technically challenging to resect than purely inflammatory disease, long-term outcomes do not differ significantly [15].

### 3.6. Strengths and Limitations

This review possesses some key strengths that contribute to the understanding of ileocecal Crohn’s disease management. Primarily, it focuses exclusively on the most recent decade of clinical research, managing to highlight the transition from a “step-up” approach towards a “surgery first” strategy. In addition, it integrates both adult and pediatric data, emphasizing potential benefits, especially in the pediatric cohort regarding growth outcomes. It is also supported by high-quality evidence from nationwide cohort studies, as well as a population of over 8000 cases. Furthermore, it provides a critical evaluation of Kono-S anastomosis, contrasting the findings from the previous decade and maintaining a more tempered approach regarding its benefits.

This study also has a certain number of limitations. Despite the high number of cases, a significant variability and heterogeneity of data exists. In addition, a substantial portion of the evidence is derived from retrospective data, which may be susceptible to selection bias. Some conclusions do not benefit from a strong grade of evidence, and while results may be clear, a more cautious tone in drawing conclusions should be advised. In addition, while pediatric results are promising, studies present a small sample size, limiting the power of the said studies. Finally, due to clinical and methodological diversity of the outcomes reported, a formal quantitative analysis was not feasible, thus requiring a narrative approach to the evidence. To mitigate the inherent selection bias observed in current retrospective registry data, future clinical research must prioritize the establishment of prospective, multicenter observational studies that utilize standardized inclusion criteria and ‘real-world’ protocols to directly compare early surgical intervention with top-down biological therapy.

## 4. Conclusions

The evidence from the past decade strongly supports the integration of surgical management as a proactive, early-line therapy for ileocecal Crohn’s disease. The long-term follow-up of the “LIR!C” trial has provided emerging evidence in terms of the surgery-first approach, demonstrating superior 10-year therapy-free remission compared to biologics. Advancements in robotic and laparoscopic techniques have significantly reduced the morbidity associated with resection. The discussion for the optimal method of anastomosis in reducing recurrence remains currently open, especially regarding the Kono-S anastomosis. Further studies are required to adequately address the risk of recurrence. In the pediatric population, the benefits of surgery extend to growth preservation and a reduction in long-term drug burden and may imply the need for early surgery to maintain adequate growth in children.

While the “mesentery question” and the optimal anastomotic technique remain areas of active research, the overall safety and efficacy of ileocecal resection are well-established. Future management of ileocecal Crohn’s disease will likely involve a personalized approach, where surgery is timed to maximize its biological and mechanical benefits, supported by minimally invasive platforms and robust postoperative medical prophylaxis. The benefits of surgical management in this decade are clear: improved long-term durability, reduced medication exposure, and a faster return to a high quality of life for both adult and pediatric patients.

## Figures and Tables

**Table 2:** Quality assessment of included studies.

Study (Year)	Study Design	Assessment Tool	Score/Risk of Bias
**Oldenburg (LIR!C) (2026) [13]**	RCT	RoB 2	Low Risk
Tyrode et al. (2024) [18]	RCT	RoB 2	Moderate (Some concerns)
**Luglio (SuPREMe-CD) (2020) [27]**	RCT	RoB 2	Low Risk
Madaffari et al. (2025) [17]	Cohort	NOS	7/9 (High)
Albert et al. (2024) [19]	Cohort	NOS	7/9 (High)
Avellenda et al. (2025) [15]	Cohort	NOS	9/9 (High)
Sarikaya et al. (2023) [22]	Cohort	NOS	9/9 (High)
Beelen et al. (2023) [21]	Cohort	NOS	8/9 (High)
Agrawal et al. (2023) [20]	Cohort	NOS	8/9 (High)
Mineccia et al. (2022) [23]	Cohort	NOS	7/9 (High)
Dreznik et al. (2023) [24]	Cohort	NOS	6/9 (Fair)
Pak et al. (2021) [25]	Cohort	NOS	7/9 (High)
Kelm et al. (2021) [26]	Cohort	NOS	6/9 (Fair)
Hirten et al. (2020) [28]	Cohort	NOS	7/9 (High)

**Table 3:** Grade of summary findings in highlighted articles.

Outcome	No. of Studies (Design)	Certainty (GRADE)	Summary of Clinical Evidence
Therapy-free Remission (Long-term)	2 (1 RCT, 1 Cohort)	MODERATE	Early ileocecal resection (ICR) provides superior 10-year therapy-free remission compared to biologics (35.8% vs. 13.2%, *p* = 0.004). Early ICR also reduces the need for postoperative medical escalation.
Risk of Reoperation (Surgical Timing)	3 (Cohort)	HIGH	Surgery within 30 days of diagnosis is associated with a 14% to 33% reduction in long-term reoperation risk compared to delayed intervention.
Postoperative Complications (MIS vs. Open)	2 (1 Meta-analysis, 1 Cohort)	HIGH	Minimally invasive surgery (MIS), specifically robotic and laparoscopic approaches, significantly reduces hospital stay and complications (14% vs. 32%) compared to open surgery.
Pediatric Growth and Nutritional Outcomes	3 (Cohort)	MODERATE	Early resection in children results in significant “catch-up” growth, with marked improvements in Z-scores for weight, BMI, and height (*p* < 0.001).
Endoscopic Recurrence (Kono-S)	4 (2 RCT, 1 Meta-analysis, 1 Cohort)	LOW	Evidence is inconsistent. While one RCT showed a dramatic reduction in recurrence, larger subsequent trials and cohorts found no significant difference compared to standard side-to-side anastomosis.
Impact of Mesenteric Management	2 (Cohort)	LOW	Current evidence suggests that radical mesenteric resection does not significantly reduce clinical or endoscopic recurrence rates.
Anastomotic Ulcer Recurrence Risk	1 (Cohort)	LOW	The presence of postoperative anastomotic ulcers is a strong predictor of clinical recurrence (aHR 3.64), highlighting the need for early endoscopic monitoring.
Recurrence in Inflammatory vs. Complicated CD	1 (Cohort)	LOW	Long-term recurrence rates are comparable between patients undergoing surgery for purely inflammatory disease versus those with complicated phenotypes.

**Table 4:** Articles focusing on early ICR.

Author et al.	Year	Article Title	Cases	Study Type and Design	Key Results (Outcomes/Safety)
Oldenburg et al. [13]	2026	Ileocaecal resection versus infliximab for ileal Crohn’s disease: 10-year follow-up of the LIR!C trial	134	Cohort study; retrospective 10-year follow-up of the LIR!C RCT.	10-year therapy-free remission: 35.8% vs. 13.2% (*p* = 0.004). Strong age-dependent benefit.
Agrawal et al. [20]	2023	Early ileocecal resection for Crohn’s disease is associated with improved long-term outcomes compared with anti-tumor necrosis factor therapy: A population-based cohort study	1279	Retrospective cohort; propensity-weighted Danish register analysis (2003–2018).	ICR associated with 33\% lower risk of composite adverse outcomes vs. anti-TNF (aHR = 0.67).
Beelen et al. [21]	2023	Impact of timing of primary ileocecal resection on prognosis in patients with Crohn’s disease	822	Cohort study; retrospective multicenter assessment.	Postoperative recurrence was not affected by timing of the ileocecal resection.
Sarikaya et al. [22]	2023	Disease course and treatment outcomes of Crohn’s disease patients with early or late surgery—A Danish nationwide cohort study from 1997 to 2015	2483	Nationwide cohort study.	Early resection had a lower cumulative risk of reoperation and a lower cumulative exposure to immunomodulators.
Kelm et al. [26]	2021	Early Ileocecal Resection Is an Effective Therapy in Isolated Crohn’s disease	103	Cohort study; retrospective single-center assessment.	Early ICR reduced the need for postoperative immunosuppressive medication.

**Table 5:** Studies focusing on evaluating Kono-S anastomosis in adult surgery.

Author et al.	Year	Article Title	Cases	Study Type and Design	Key Results (Outcomes/Safety)
Baloyiannis et al. [16]	2024	The Reduction in Anastomosis-Related Morbidity Using the Kono-S Anastomosis in Patients with Crohn’s Disease: A Meta-Analysis	913	Meta-analysis; 8 studies evaluating the Kono-S hand-sewn anastomosis.	Reduced leak risk (OR = 0.34) and reoperation (OR = 0.12). No change in overall morbidity.
Tyrode et al. [18]	2024	KONO-S Anastomosis Is Not Superior to Conventional Anastomosis for the Reduction in Postoperative Endoscopic Recurrence in Crohn’s Disease	366	Randomized controlled trial; multicenter prospective randomized study.	No significant difference in 12–18 m endoscopic recurrence (31.6% Kono-S vs. 33.3% SSA).
Alibert et al. [19]	2024	Does Kono-S Anastomosis Reduce Recurrence in Crohn’s Disease Compared with Conventional Ileocolonic Anastomosis? A Nationwide Propensity Score-matched Study from GETAID Chirurgie Group [KoCoRICCO Study]	433	Propensity score analysis.	Kono-S did not reduce risk of endoscopic recurrence ≥ i2 compared to SSA (47.5% vs. 44.3%).
Luglio et al. [27]	2020	Surgical Prevention of Anastomotic Recurrence by Excluding Mesentery in Crohn’s Disease: The SuPREMe-CD Study—A Randomized Clinical Trial	113	Randomized controlled trial; Kono-S vs. conventional stapled side-to-side.	Kono-S group showed significantly lower endoscopic recurrence (*p* < 0.05) and clinical recurrence at 12–18 months.

**Table 6:** Studies focusing on surgical modalities and technical advancements.

Author	Year	Cases	Study Type and Design	Key Results (Outcomes/Safety)
Flaifel et al. [14]	2025	5760	Meta-analysis; systematic review of RIR vs. LIR/OR	Robotic approach had longer operative times (226 min) but shorter hospital stays and fewer complications than open surgery.
Mineccia et al. [23]	2022	326	Retrospective cohort	Resection of the mesentery does not reduce endoscopic and recurrence rate.
Pak et al. [25]	2021	348	Cohort study; propensity -score-matched comparison of LICR vs. OICR	LICR reduced complications (14\% vs. 32\%) and hospital stay (8 days vs. 13 days).
Luglio et al. [27]	2020	113	Randomized controlled trial; Kono-S vs. conventional stapled side-to-side	Kono-S group showed significantly lower endoscopic recurrence (*p* < 0.05) and clinical recurrence at 12–18 months.
Hirten et al. [28]	2020	182	Retrospective cohort	Multivariable analysis: anastomotic ulcers were associated with disease recurrence ([aHR] 3.64; 95% CI, 1.21–10.95; *p* = 0.02); 66 subjects with anastomotic ulcers underwent a second colonoscopy, with 31 patients (79.5%) having persistent ulcers independent of medication escalation.

## Data Availability

No new data were created or analyzed in this study.

## Supplementary Materials

The following supporting information can be downloaded at https://www.mdpi.com/article/10.3390/medicina62050949/s1: Table S1: PRISMA 2020 Checklist [48].
